# Does the national health insurance scheme in Ghana reduce household cost of treating malaria in the Kassena-Nankana districts?

**DOI:** 10.3402/gha.v7.23848

**Published:** 2014-05-13

**Authors:** Maxwell Ayindenaba Dalaba, Patricia Akweongo, Raymond Aborigo, Timothy Awine, Daniel Kweku Azongo, Prosper Asaana, Frank Atuguba, Abraham Oduro

**Affiliations:** 1 Navrongo Health Research Centre, Navrongo, Ghana; 2 School of Public Health, University of Ghana, Accra, Ghana; 3 Global Public Health, Monash University, Malaysia

**Keywords:** national health insurance, treatment seeking, malaria, cost, Kassena-Nankana district, Ghana

## Abstract

**Introduction:**

The Government of Ghana introduced the National Health Insurance Scheme (NHIS) in 2003 to replace out-of-pocket (OOP) payment for health services with the inherent aim of reducing the direct cost of treating illness to households.

**Objective:**

To assess the effects of the NHIS in reducing cost of treating malaria to households in the Kassena-Nankana districts of northern Ghana.

**Methods:**

We conducted a cross-sectional survey between October 2009 and October 2011 in the Kassena-Nankana districts. A sample of 4,226 households was randomly drawn from the Navrongo Health and Demographic Surveillance System household database and administered a structured interview. The costs of malaria treatment were collected from the patient perspective.

**Results:**

Of the 4,226 households visited, a total of 1,324 (31%) household members reported fever and 51% (675) reported treatment for malaria and provided information on where they sought care. Most respondents sought malaria treatment from formal health facilities 63% (424), with the remainder either self-medicating with drugs from chemical shops 32% (217) or with leftover drugs or herbs 5% (34). Most of those who sought care from formal health facilities were insured 79% (334). The average direct medical cost of treating malaria was GH¢3.2 (US$2.1) per case with the insured spending less (GH¢2.6/US$1.7) per case than the uninsured (GH¢3.2/US$2.1). The overall average cost (direct and indirect) incurred by households per malaria treatment was GH¢20.9 (US$13.9). Though the insured accounted for a larger proportion of admissions at health facilities 76% (31) than the uninsured 24% (10), the average amount households spent on the insured was less (GH¢4/US$2.7) than their uninsured counterparts (GH¢6.4/US$4.3). The difference was not statistically significant (*p*=0.2330).

**Conclusion:**

Even though some insured individuals made OOP payments for direct medical care, there is evidence that the NHIS has a protective effect on cost (outpatient and in-patient) of malaria treatment.

Malaria is the leading cause of morbidity and mortality in sub-Saharan Africa. Malaria treatment costs in Africa accounts for about 1.3% annual reduction in economic growth (1). Productivity loss due to a case of malaria in an adult in endemic areas of Africa ranges from 1 to 6 days, causing a considerable loss of productive time (1).

In Ghana, malaria accounts for about 38% of outpatient attendance, 36% of all admissions, and 33% of all deaths among children aged under 5 years (2). The number of days lost per episode of malaria is estimated at 9 days for the patient and 5 days for the caretakers (3). Malaria causes a loss of about 10.6% Disability Adjusted Life Years (DALYs) and costs 6% of gross domestic product (GDP) annually in economic burden (4). In northern Ghana, the estimated average cost of treating malaria ranges from US$6.39 to 15.79 per case (3, 5). The consequences of cost of treating malaria can alter health-seeking behaviors and affect the demand for formal health services, thus shifting care to the informal and often unregulated providers (3).

The Ghana National Malaria Control Programme (NMCP) aims at reducing the malaria disease burden by 75% by 2015. Current initiatives to achieve this target include indoor residual spraying, use of insecticide-treated bed nets, artemisinin-based combination therapies, and intermittent preventive treatment in pregnant women (2).

Financing of health services has been a challenge in Ghana's history. After Ghana gained independence in 1957, public health services were provided free through tax revenue (6, 7), but by the 1980s, the system became financially unsustainable. Consequently, the government introduced user fees where individuals paid out-of-pocket (OOP) for health services. However, the user fees brought about challenges of equity in access to health care especially for the poor (8) and decreased the utilization of health care services at the public health facilities (7, 9). By 1990, Ghana began to consider community-based health insurance schemes (CBHIS) as an option for financing health care. The system was piloted in some selected communities and subsequently adopted as the health financing policy for the country. The National Health Insurance Scheme (NHIS) was then established in October 2003 under Act 650 (10). The primary goal of the NHIS is to improve access to and quality of basic health care services in Ghana through the establishment of mandatory district-level mutual health insurance schemes. The system aims to replace OOP payments for health services and to provide financial protection against high costs of health care or catastrophic spending (11).

The NHIS covers about 95% of diseases including malaria. Children below 18 years, the aged (70 + ) in the informal sector, and indigents are exempted from paying premiums. All adults who do not contribute to the national social security scheme (mostly the informal sector workers) have to pay an annual minimum premium of GH¢7.2 (US$4.8). On the contrary, the premiums for workers who contribute to social security (mostly formal sector workers) are deducted directly from their social security contributions (12). The NHIS is financed by a 2.5% National Health Insurance Levy (NHIL) on selected goods and services, a 2.5% Social Security and National Insurance Trust (SSNIT) deductions from the formal workers, premiums from the informal workers and non-SSNIT contributors, and government budget allocations. The NHIS national coverage is not encouraging since only 34% of the population had valid membership cards as at the end of December 2012 (13).

Despite the operation of the NHIS since 2003, it remains unclear if the scheme has succeeded in reducing the costs of malaria treatment to households. Moreover, there has not been any published study on the effects of the NHIS on OOP payments in the Kassena-Nankana district (one of the poorest districts in Ghana). This study therefore assessed the effects of the NHIS in reducing cost of treating malaria to households in the Kassena-Nankana district of northern Ghana. In the study, we used the patient viewpoint and included two main costs categories: direct and indirect costs (3, 14). The data presented are part of a wider phase 4 study aimed at assessing the effectiveness and safety of antimalarial drugs in Africa.

## Methods

### Study site

The study was carried out in the Kassena-Nankana district of northern Ghana. Even though the district has been divided into two – the Kassena-Nankana East and West districts – for the purposes of this study, we shall refer to the district by its former name – the Kassena-Nankana district. The Kassena-Nankana district has a population of 152,000 people and covers an area of about 1,675 km^2^ (15). The district is relatively dry between October and April and wet between May and September. The main occupation is subsistence agriculture.

There are two main ethnic groups: the Kassena who form about 49% of the district's population and the Nankana who constitute about 46% of the population. The remaining 5% is made up of minority tribes, mainly Builsa and migrants belonging to other ethnic groups. Despite the linguistic differences, the population is, in many respects, homogenous, with a common culture (16).

The district has a hospital located in Navrongo that serves as a referral point, seven health centers, one private clinic and 27 Community-Based Health Planning and Services (CHPS) compounds. These CHPS compounds have resident Community Health Officers (CHOs) offering door-to-door services to community members (17, 18). There are two community clinics jointly run by the Catholic Diocesan Development Office and the District Health Administration. A number of traditional healers and itinerant drug vendors provide informal health care within communities. Financing health care in the district is through the Kassena-Nankana district mutual health insurance scheme and OOP payments. About 50% of the population who have enrolled into the scheme have valid membership cards to access health care from accredited providers.

The Kassena-Nankana district has a Health and Demographic Surveillance System (HDSS) run by the Navrongo Health Research Centre. As part of the Navrongo HDSS activities, households are routinely visited – three times a year – to update information on pregnancies, births, morbidity, mortality, migration, marriages, vaccination coverage, and other vital socio-demographic variables (15).

### Study design and data collection

A cross-sectional survey design was used to collect data from households between October 2009 and October 2011. We interviewed household members reporting fever or malaria irrespective of whether the patient sought treatment or not. We obtained data on socio-demographic characteristics, insurance status, treatment-seeking patterns and direct and indirect costs of malaria. Insurance status was confirmed by physically verifying the validity of insurance cards from household members.

### Sample size

We assumed conservatively that at least 6% of the population will have had a fever event 2 weeks prior to the interview date and that about 50% of the population with fever gained access to an official point of ACT provision within 24 h. We accounted for clustering within households by using a design effect of 1.5. We further allowed a 10% drop-out rate to achieve a sample size of 4,226 households.

### Ethical considerations

Extensive community entry activities were carried out through durbars with chiefs and elders throughout the district before commencement of the study. Discussions on the study were also held on a local community radio in three languages – English, Kasem, and Nankani. The study protocol was reviewed and approved by the Ghana Health Service Ethics Committee, the Kintampo Health Research Centre Institutional Review Board, and the Navrongo Health Research Centre Institutional Review Board. Also, individual written informed consent was obtained from respondents before being interviewed.

### Data processing and analysis

Data were double entered into Epidata 3.1 and transferred to Stata 11.0^©^ for analysis. We used descriptive statistics showing mean values to summarize direct and indirect costs. A *t*-test (for continues variables) and Chi-square test (categorical variables) were used to determine if there were any significant differences between the insured and uninsured on variables of interest. The focus was on descriptive associations only.

We could not validate the insurance status of 432 respondents; hence, they were not included in the analysis. Thus, a total of 892 insurance statuses of respondents were determined and included in the analysis.

Age of patients was captured as continuous variables, but during the analysis, we grouped them into four categories: under 5 years, 5–17 years, 18–69 years, and 70 years and above.

The types of health care providers that patients sought care from were grouped into three categories: formal health facilities (i.e. hospital, clinics, community health compound and health centres), chemist/pharmacy shops, and home-based care (left over drugs and traditional care).

Direct and non-direct medical (direct costs) costs were estimated by summing the costs and means calculated. The direct medical costs covered OOP payments for drugs, consultations, laboratory tests, and direct non-medical costs included special foods for patients and transportation to and from health care providers.

Indirect cost (productivity lost) was estimated by multiplying the number of days lost due to malaria by the daily wage for year 2011 (GH¢3.73). This was calculated for both the patient and the caretaker who were actively engaged in an income-generating activity prior to the illness. In-patient or admission was defined as those who were detained in a health facility for malaria for longer than 12 h. In-patient costs were therefore the sum of medical costs, laboratory costs, and bed costs during the admission.

The data for assessing the socio-economic status (wealth) of households is collected through the Navrongo Health and Demographic Health Surveillance System (15). This database has information on all households in the district. We linked the respondents of our study to the NHDSS household socio-economic data to generate the household wealth index using Principal Component Analysis Technique. The wealth or Socio-economic status (SES) of the household was measured in terms of assets and possessions using principle component analysis (PCA). These included type of material for walls, roofing material, cooking utensils, toilet facility, source of drinking water, and cooking fuel. Household possessions included bicycle, motorbike, car, radio, bed, sewing machine, tape player, TV, DVD, mobile phone, refrigerator, cattle, sheep, goat, pig, and donkey. Households were then assigned to five quintiles as used in other studies (8, 19, 20). These quintiles described wealth levels and represented it as least poor, less poor, poor, very poor, and the poorest. About 99 households could not be linked with the Navrongo HDSS household assets database and therefore could not be classified.

## Results

### Background characteristics

Of the 4,226 sampled households for the study, a total number of 1,324 (31%) respondents reported fever during the recall period and were interviewed. The insurance status was determined for a total of 892 (67%) respondents, of which 438 (49.1%) were insured (had valid health insurance card) and 454 (50.9%) were not insured. The average age of respondents was 22 years. About 59% (526) were children under 18 years (representing the exempt group), 38% (336) were adults aged between 18 and 69 years (representing the working group that pays premiums), and 3.3% (30) were aged 70 years and above (representing the aged exempt group). In total, 53% (473) were females. About 70% (624) of the respondents were unemployed, 17.4% (155) were farmers, 10% (90) were artisans, and 2.6% (23) were employed in the formal sector. About 25% (329) of respondents were from the least poor households whereas 17% (141) were from the poorest households (Table 1).

**Table 1:** Background characteristics for insured and uninsured respondents

Variable	Insured	Uninsured	Total	*p*
Age				0.004
under 5 years	181 (41.3%)	151 (33.3%)	332 (37.2%)	
5–17 years	87 (19.9%)	107 (23.6%)	194 (21.8%)	
18–69	149 (34%)	187 (41.2%)	336 (37.7%)	
70 +	21 (4.8%)	9 (2%)	30 (3.3%)	
Total	438 (100%)	454 (100%)	892 (100)	
Sex				0.008
Male	186 (42.5%)	233 (51.3%)	419 (47%)	
Female	252 (57.5%)	221 (48.7%)	473 (53%)	
Total	438 (100%)	454 (100%)	892 (100)	
Occupation				0.008
Farmer	57 (13%)	98 (21.6%)	155 (17.4%)	
Formal worker	13 (3%)	10 (2.2%)	23 (2.6%)	
Artisan	44 (10%)	46 (10.1%)	90 (10%)	
Unemployed	324 (74%)	300 (66.1%)	624 (70%)	
Total	438 (100%)	454 (100%)	892 (100%)	
Wealth				0.000
Least poor	82 (20.6%)	122 (28.3%)	204 (24.6%)	
less poor	61 (15.3%)	96 (22.3%)	157 (18.9%)	
Poor	81 (20.4%)	92 (21.4%)	173 (20.9)	
very poor	78 (19.6)	76 (17.6%)	154 (18.6%)	
Poorest	96 (24.1%)	45 (10.4%)	141 (17%)	
Total	398 (100%)	431 (100)	829 (100%)	

### Health care–seeking behavior

Out of the 1,324 respondents who reported fever, 51% (675) reported treatment for malaria and provided information on where they sought care. Of those who provided information on treatment source, 52% (354) were insured. About 63% (424) sought care at a formal health facility, 32.2% (217) bought drugs from chemical shops, and 5% (34) were treated at home using herbs and leftover drugs (Table 2). About 79% (334) of the patients who sought care at the formal health facilities were insured.

**Table 2:** Source of malaria treatment

Variable	Insured	Uninsured	Total
Source of care	Frequency	Frequency	Total (Frequency)
Home-based care	9 (2.5%)	25 (7.8%)	34 (5%)
Formal provider	334 (94.4%)	90 (28%)	424 (62.8%)
Chemical shops	11 (3.1%)	206 (64.2)	217 (32.2%)
Total	354 (100%)	321 (100%)	675 (100%)

### Direct and indirect costs of malaria

Table 3 presents direct and indirect costs due to malaria. Out of the 675 respondents who sought care, only 199 (29.5%) incurred direct medical costs during malaria treatment, and the rest reported zero direct medical cost. The average direct medical cost was GH¢3.2 or US$2.1 per treatment. Out of the number who incurred direct medical cost (199) about 97% (192) were uninsured patients. The average cost incurred on direct medical cost was higher among the uninsured (GH¢3.2/US$2.1) than the insured (GH¢2.6/US$1.7), though the difference was not statistically significant (*p*=0.2874). The average direct non-medical cost was GH¢2.3/US$1.5. Therefore, households spent an average of GH¢5.5/US$3.7 on direct cost (medical and non-medical) per malaria episode. The average indirect cost incurred by households due to the malaria episode was GH¢15.4/US$10.

**Table 3:** Direct and indirect costs of malaria outpatient care

Cost		Insured		Uninsured		Total		
				
category	Sub-category	GH¢ (US$)	Frequency	GH¢ (US$)	Frequency	GH¢ (US$)	Frequency	*p*
Direct cost	Direct non-medical	GH¢2.1(US$1.4)	27	GH¢2.5 (US$1.7)	23	GH¢2.3 (US$1.5)	50	0.2421
	Direct medical cost	GH¢2.6 (US$1.7)	7	GH¢3.2 (US$2.1)	192	GH¢3.2 (US$2.1)	199	0.1925
Total direct cost	Non-medical plus medical cost	GH¢4.7 (US$3.1)	34	GH¢5.7 (US$3.8)	215	GH¢5.5 (US$3.7)	249	0.1352
Indirect cost	Indirect cost	GH¢14.9 (US$9.9)	257	GH¢15.9 (US$10.6)	226	GH¢15.4 (US$10.3)	483	0.1540
Total cost	Direct plus indirect costs	GH¢19.6 (US$13.1)	291	GH¢21.6 (US$14.4)	441	GH¢20.9 (US$13.9)	732	0.0372

The overall average cost (direct and indirect cost) incurred by households per malaria treatment was GH¢20.9/US$13.9. Average cost incurred in the treatment of malaria by the insured was less (GH¢19.6/US$13.1) than their uninsured counterparts (GH¢21.6/US$14.4).

### Hospitalization

Less than 5% (41) of patients were admitted or detained in a health facility for more than 12 h due to malaria. More insured patients 76% (31) were hospitalized than the uninsured 24% (10). The average number of days spent by patients hospitalized was 2 days with the insured spending more days (2 days) than the uninsured (1 day). The average amount households spent on patients who were admitted was GH¢6.4(US$4.3). Households spent less on insured inpatients (GH¢4/US$2.7) than on uninsured inpatients (GH¢6.4/US$4.3), though the difference was not statistically significant (*p*=0.2330).

### Number of days lost due to malaria

The average number of days a malaria episode prevented patients from working was 5 days for patients and 3 days for the caregivers. There was no difference in the number of days lost due to malaria between the insured and the uninsured. When the analysis was done to exclude children, the number of days lost still remained at 5 days. The number of school days lost by patients attending school due to malaria was 4 days. There was no difference on the number of school days lost between the insured patients and the uninsured patients. The average number of days after the onset of malaria that the first treatment source was visited was 3 days, with a significant difference (*p*<0.05) between the insured (3 days) and the uninsured (2 days).

### Limitations of the study

Recall bias is a possible limitation, particularly in cases that respondents did not produce receipts on expenditure but only verbal reporting. This is a common problem reported in many studies. However, with the effective training of data collectors on probing mechanisms, this was minimized. Another limitation of the study is the generalization of the results outside the study district. The study district may differ from other districts due to differences in malaria endemicity, access to health facilities, access to antimalarials and other direct and direct inputs.

Another limitation is the use of PCA for the assessment of wealth/SES. We used the same asset lists for semi-urban and rural areas, which could have biased the households’ wealth status, constituting one of the limitations of PCA (21). Values differ by area, for instance, the value for donkey does not have the same utility (value) for a farmer living in a rural area and a teacher residing in a semi-urban area. Notwithstanding this, with the difficulties of obtaining accurate income data, PCA is more appropriate and this has been applied in other studies like the Ghana Demographic Health Surveys (GDHS) (8, 19, 20).

Despite these limitations, the large sample size, and the use of the NHDSS, the results are reliable and provide relevant evidence on the economic burden of malaria to households and the effects of NHIS on OOP for malaria treatment.

## Discussion

This study revealed that most malaria patients do not self-treat but rather seek care from formal health facilities such as government and private health facilities. This health-seeking pattern contrasts with a similar study in the same setting where many households resorted to self-medication (5). The NHIS provides free consultations, laboratory diagnoses, and drugs for patients who are enrolled into the scheme. The trend of seeking formal care may be attributed to the enrolment status of patients as most people who sought care from formal health facilities were enrolled into the NHIS. The effect of NHIS in increasing care seeking in formal health care facilities has been reported in other studies (6, 22–24).

A study by Akazili et al. reported a lower average cost for treating an episode of malaria than the current study. The key reason for this trend could be the change in the first line treatment for malaria as well as the encouragement of the use of diagnostic tests (microscopy or Rapid Diagnostic Tests) to confirm malaria before offering treatment. Chloroquine which was the first line drug was GH¢0.01 (US$0.007) while the current first line Artesunate-Amodiaquine is GH¢2 (US$1.3) per adult in a government health facility. Also, medicines such as those for treating malaria in public health facilities have been subsidized by the government, therefore making the prices quite low. For instance, the price of Artersunate-Amodiaquine is GH¢5(US$3.3) in the pharmacies. In spite of this subsidy, malaria treatment can still be described as high for the predominantly poor population in the study area.

Again, most of the patients who made OOP payments for direct medical care were uninsured (97%) and they paid more (GH¢3.2/US$2.1) on average than the insured (GH¢2.6/US$1.7). This is consistent with findings from some studies conducted in Ghana (25) and Senegal (24). Even though the difference on OOP between insured and uninsured was not statistically significant, it is important to note that the people in the Kassena-Nankana district are among the poorest in Ghana and therefore any marginal difference in expenditure affects attitude towards treatment. In addition, expenditure on malaria treatment may not only stretch the already tight budgets of households, especially poorer households, but also push them into the poverty trap and contribute to lowering the standard of living. The situation is exacerbated in cases of multiple malaria episodes within a household for a season.

Our study showed that the indirect cost per episode of malaria was 2.8 times higher (GH¢15.4/US$10.3) than the direct cost (GH¢5.6/US$3.7). Similar findings where indirect costs were found to be higher than direct costs have been reported in related studies (5, 26, 27). The NHIS does not cover indirect costs and therefore this cost is completely borne by the patient irrespective of their NHIS status. This has the potential to derail the benefits of the scheme.

The number of days lost due to malaria by patients and caretakers was 5 and 3 days, respectively, which is similar to results of a study conducted in Ethiopia in which the number of days lost due to malaria ranged from 1 to 6 days (1) but different from an earlier study conducted in Ghana where the average number of days lost due to malaria ranged from 5 to 9 days (3). The number of days lost by students (4 days) is worrying as it negatively affects school performance and becomes a challenge to human development. In addition, though the productivity days lost due to malaria has declined over the period, the current level is still worrying. Given that the main economic activity of most people in the district is farming and malaria transmission is intense during the farming period, workdays lost adversely affects household production, GDP, and ultimately economic growth and development.

It was expected that the insured would respond to treatment promptly because of a reduction in cost to access; however, this was not the case. One plausible reason could be due to longer waiting time, verbal abuse, and poor quality of treatment that the insured experience as compared to the uninsured (25). The insured only arrive at health facilities when their health condition has deteriorated, thus accounting for the huge proportion (76%) that reported being hospitalized. Nevertheless, membership into the NHIS was associated with lower in-patient cost. This corroborates with other studies in which mutual health organization coverage for in-patient care reduced OOP expenditures for hospitalization in both Ghana and Senegal (24).

## Conclusions

Even though some insured made OOP payments for direct medical care, there is evidence that the NHIS has a protective effect on cost (outpatient and in-patient) of malaria treatment. The burden of malaria can adversely affect households and force poorer households to substitute their productive capital for health care thereby pushing them into a poverty trap. Innovative approaches to increase enrolment for the poor are urgently needed to speed up universal coverage and reduce the financial burden of diseases.
